# The relationship between anxiety and depression under the pandemic: The role of life meaning

**DOI:** 10.3389/fpsyg.2022.1059330

**Published:** 2022-11-28

**Authors:** Daniel T. L. Shek, Wenyu Chai, Lindan Tan

**Affiliations:** Department of Applied Social Sciences, The Hong Kong Polytechnic University, Kowloon, Hong Kong SAR, China

**Keywords:** anxiety, life meaning, spirituality, depression, comorbidity, positive youth development

## Abstract

**Introduction:**

COVID-19 is a stressor creating much anxiety for the general public, such as anxiety related to possible infection, social distancing, financial strain and uncertainty. As the scientific literature shows that there is an intimate relationship between anxiety and depression, it is important to ask whether anxiety is related to depression under the pandemic and whether spirituality indexed by life meaning can moderate the relationship between anxiety and depression. According to theories highlighting the importance of life meaning, relative to people with a higher level of life meaning, the relationship between anxiety and depression would be stronger in people with a lower level of life meaning.

**Methods:**

Empirically, we collected data in two waves (i.e., before and after the first wave of COVID-19, respectively) from 4,981 adolescents recruited in Sichuan, China. Then, the 41-item “Screen for Child Anxiety Related Emotional Disorders” was employed to measure anxiety symptoms, 20-item “Center for Epidemiological Studies-Depression Scale” was utilized to examine depression symptoms, and the “Spirituality Subscale of the Chinese Positive Youth Development Scale” for assessing life meaning.

**Results:**

We found that anxiety significantly predicted depression at each wave and across time. Second, controlling for Wave 1 depression scores, results showed that a drop in Wave 1 anxiety predicted a drop in depressive symptoms over time. Regarding the relationship between meaning in life and depression, spirituality indexed by meaning in life negatively predicted depression at each wave and over time, and predicted change in depression across time. Finally, multiple regression analyses showed that life meaning moderated the predictive effect of anxiety on depression.

**Discussion:**

The findings support the thesis that spirituality serves as a protective factor for psychological morbidity in Chinese adolescents. The study also suggests the importance of helping adolescents to develop life meaning under COVID-19.

## Introduction

Anxiety is a negative emotional state that is prevalent in different stages of the life cycle, especially in childhood and adolescence (Blumberg and Izard, 1986; Cohen et al., 2018). There is also an intimate link between anxiety and other negative emotional states, particularly depression. While anxiety and depression have similar emotional profiles (Clark and Watson, 1991; Brady and Kendall, 1992), they are different in terms of several areas. Blumberg and Izard (1986) pointed out that while fear and apprehension are dominant in anxiety, sadness and lack of energy are central features of depression. Higgin et al.’s (Higgins et al., 1985) self-concept discrepancy theory also highlights that depression is primarily related to dejection whereas anxiety is characterized by agitation. According to Beck and Clark (1988), the perceived physical or psychological threat is central to anxiety whereas the depressive state emphasizes loss or deprivation. Watson et al. (1995) further proposed that “anhedonia” is a central feature of depression whereas “hyperarousal” is the prime attribute of anxiety disorders.

Empirically, consistent results across comorbidity studies suggest the co-existence of anxiety and depression at the same time (e.g., Almeida et al., 2012). Under COVID-19, the reported combined rate of anxiety and depression was 12.4%, with incidence rates of 14% and 19% for anxiety and depression, respectively, among 500 participants in Hong Kong society (Choi et al., 2020). Axelson and Birmaher (2001) concluded that approximately 10%–15% of adolescents reporting anxiety symptoms also report depressive disorders and around 25%–50% of youth experiencing depression have a comorbid anxiety disorder. As early as 1988, the World Health Organization initiated an international study involving 14 countries with concerns about mental health in primary care, discovering that “nearly half of the cases of depression and anxiety appeared in the same patients and at the same time” (Sartorius et al., 1996, p. 40). In New Zealand, according to a birth cohort (*N* = 1,037), Moffitt et al. (2007) found that while for 32% with anxiety disorders, depression preceded or coincided with anxiety disorder, anxiety occurred before or simultaneously with the onset of depression among up to 37% of depression cases.

In this study, we explored several issues using a longitudinal dataset collected from young people in mainland China before and after school lockdown in the first wave of the pandemic in 2019–2020. These include the predictive effect of anxiety on depression; the predictive effect of life meaning on depression; and the moderating effect of life meaning on the relationship between anxiety and depression.

### Relationship between anxiety and depression

There are continuous debates on the causal relationships (i.e., anxiety contributes to depression; depression results in anxiety) and comorbidity involved in anxiety and depression (Cohen et al., 2018). On the one hand, there are several conceptual models proposing that anxiety is an antecedent of depression. Barlow (2000) proposed the triple vulnerabilities model of anxiety disorders. In this model, it was hypothesized that “generalized biological vulnerability” combined with “generalized psychological vulnerability” (e.g., weaker sense of predictability and control) and “specific psychological vulnerabilities” rooted in special early risk learning experiences would lead to stress (p. 1257), which would further lead to generalized anxiety and depression. With reference to genetic factors, stressful life events, anxiety traits and depression, Sandi and Richter-Levin (2009) proposed that genetic risk factors and early experiences serve crucial roles during the evolution of high anxiety trait neuroticism, which is the primary etiology for the anxiety disorder incidence leading to the development of depression. To elucidate the developmental trajectories and relational patterns of internalizing problems over childhood and adolescence, Cohen et al. (2018) hypothesized a “heterotypic discontinuity” model proposing that childhood anxiety shapes adolescent depressive symptomology. While “heterotypic continuity” arises when a symptom expression sets the stage for one new form of psychopathology, “discontinuity” refers to the same symptom functioning differently at various stages of progression. In their study, they found that while child anxiety predicted adolescent anxiety, child anxiety (and child depression) predicted adolescent depression. In another theoretical framework, Cyranowski et al. (2000) explained “depressogenic vulnerabilities” in women. It maintains that a combination of “insecure parental attachments,” “anxious/inhibited temperament,” and “low instrumental coping skills” for independence (and interaction) put individuals at risk for “difficult adolescent transition,” then resulting in “anxiety.” Concurrently, female-specific “hormonal changes at puberty,” “pubertal intensification in affiliative need,” along with “female gender socialization” lead females to greater vulnerability throughout this process, creating a stronger “depressogenic diathesis” with anxiety at its core. Subsequently, high-risk females who possess high anxiety accompanied by “elevated affiliative focus, low attachment security, and low instrumentality” are predisposed to depression when coping with “negative life events” (p. 24).

On the other hand, there are models proposing that depression is an antecedent of anxiety, although the number of such theories is few. In an early study, Neale and Kendler (1995) suggested that major depression might cause generalized anxiety disorder (i.e., increased anxiety because of higher depression liability). Weeks et al. (2009) also formulated a cognitive model on social anxiety highlighting that “fear of evaluation and depressive cognitions lead to social anxiety and submissive withdrawal” (p. 375).

Finally, there are views suggesting the comorbidity of anxiety with depression. Brady and Kendall (1992) proposed that anxiety symptoms are at a greater likelihood of occurring in conjunction with depression rather than in isolation, and that they highly overlap. Similarly, Clark and Watson (1991) argued that syndromes of anxiety and depression possess common nonspecific components (i.e., “negative affectivity”), such as upset, distress and general maladjustment, while the two are distinguished by “physiological hyperarousal” particular to anxiety and “absence of positive affect” (e.g., anhedonia) typical of depression (p. 316). Likewise, Neale and Kendler (1995) proposed the co-morbidity of generalized anxiety and major depression could be contributed by a cluster of shared genetic and environmental risk factors (i.e., correlated liabilities model). Consistent with the shared etiology hypothesis, Alloy et al. (1990) proposed a helplessness-hopelessness perspective for anxiety and depression disorders to explain the high comorbidity of the two domains. When confronted with negative life events, individuals with pessimistic inferential styles are much more inclined to generate a feeling of helplessness that would increase their likelihood of developing hopelessness, which is a substantial cause of anxiety and depression. For the comorbidity of anxiety with depression in young people, Seligman and Ollendick (1998) proposed four possible explanations. First, it is due to conceptual overlap such as the symptoms involved. Second, they are different indicators underlying a single construct. Third, overlaps in risk factors lead to the observation of comorbidity. Finally, anxiety increases depression risk in young people.

### Empirical evidence on the predictive relationships between anxiety and depression

There is research evidence supporting the claim that anxiety is a precursor of depression. Pine et al. (1998) showed adolescent anxiety or depression significantly predicted an enhanced peril of depression in adulthood by approximately 2 to 3 fold. Based on a birth cohort of 1,265 New Zealand children, Woodward and Fergusson (2001) revealed a positive significant association between reported anxiety during teenage years and the later onset of anxiety disorders, major depressive disorder, and drug abuse. Chaplin et al.’s (2009) longitudinal survey in five middle schools also showed a predictive contribution of anxiety and worry to depressive symptoms one year later, which was more intense in girls than boys. Assessing Norwegian children (*N* = 1,439), Aune and Stiles (2009) showed that while preliminary depressive signs were not observed to predict future social anxiety, initial social anxiety did predict the evolution of depression. Similarly, tracking Swedish middle-aged and older twins (*N* = 1,391), Wetherell et al. (2001) revealed that despite the fact that depression and anxiety were strongly associated and anxiety symptoms led significantly to the progression of depression over time, the opposite causal direction failed to hold.

Studies also showed the predictive relationship of anxiety with depression in clinical studies. Beesdo et al. (2007) discovered that the cumulative incidence of social anxiety disorder was 11.0% and subsequent major depressive episode or dysthymia was 27.0% among 3,021 young adults in Germany. According to Parker et al. (1999), anxiety manifested as social avoidance or inhibition preceded “early-onset non-melancholic major depression” (p. 11) particularly readily, with the character of anxiety established as a risk role. Bittner et al. (2007) showed that childhood overanxious disorder significantly predicted both overanxious disorder and depression in adolescence. Coelho et al. (2011) suggested that high-level antenatal generalized anxiety disorder was detected as an independent predictive factor for postnatal depression. Among the 250 patients in primary care struggling with both affective disorders and physical pain who live in the United States, Bair et al. (2013) revealed that baseline generalized anxiety disorder significantly predicted depression severity after 12 months. Similarly, Starr and Davila (2012) revealed that, at various time lags, daily anxiety anticipated the subsequent emergence of the depressed mood, whereas they simultaneously found that depressed mood consistently failed to forecast later anxiety.

Under COVID-19, Vowels et al. (2022) found that personal attachment anxiety significantly predicted individual depression, while their partner’s attachment style did not predict the worsening of one’s mental health. In contrast, Beesdo et al. (2007) argued that not only the severity of social anxiety disorder predicted individual subsequent depression, but the predictive effect of parental anxiety was also significant. Ranney’s et al. (2021) research additionally suggested that parental anxiety was significantly categorized as one of the critical risk contributors to their children’s depression progression. There are also studies examining mediating factors in the significant linkage between anxiety and depression, including non-acceptance in intimate relationships (Jacobson and Newman, 2016), anhedonia (Winer et al., 2017), and avoidance (Jacobson and Newman, 2014).

On the other hand, there are few studies suggesting that depression predicts anxiety. Kim-Cohen et al. (2003) also showed that individuals suffering from adult anxiety were considered probable with a depression history during adolescence. Rallis et al. (2014) found among 214 pregnant women in Australia that an increase in depression in early pregnancy was predictive of elevated stress and anxiety in late pregnancy. As opposed, Schiffer et al. (2008) argued that negative affectivity and social inhibition, rather than depressive symptoms, predicted clinical anxiety based on the sample of Dutch patients with systolic chronic heart failure.

Besides the above two categories of evidence, there are studies suggesting the bidirectional influences between anxiety and depression, although the intermediate latent trajectories and mechanisms of such influences are as yet less clear (Olino et al., 2010). Based on a 3-year longitudinal study of elementary school children (*N* = 330) and their parents (*N* = 228), Cole et al. (1998) found that both child-and parent-reported anxiety positively predicted a slight but significantly increased childhood depression over time, yet unexpectedly discovered that parent-reported child depression negatively predicted anxiety, implying that children whose parents reported depression had lower anxiety levels subsequently. Echoing this observation, Snyder et al. (2009) identified that teacher-rated child anxiety symptoms from children aged 5.3 to 6.8 years promoted later depressive symptoms, whereas depressive symptoms suppressed later anxiety from children aged 6.8 to 9.3 years. After performing a meta-analysis encompassing 66 studies, Jacobson and Newman (2017) reached the conclusion that anxiety and depression acted as bidirectional risk factors toward each other longitudinally.

### Life meaning and psychological well-being

Life meaning plays an important role in existential psychology. In contrast to the behaviorists’ view that human beings are passive organisms responding to environmental stimuli and the psychoanalysts’ belief that human beings are susceptible to the influence of inner instincts, existential psychologists assert that individuals are capable of making authentic choices within their freedom and constraints of the reality. With particular reference to Frankl (1959), he posited that human beings are not passively responding to the environment and are not motivated by the “will to pleasure” (i.e., psychoanalytic focus on hedonic drives). Instead, life meaning (i.e., “will” to meaning) plays an important role in human behavior. The basic thesis is that when there is no meaning in an individual (i.e., existential vacuum), psychopathologies come in to fill the psychological vacuum. According to Crumbaugh and Maholick (1964), life purpose or life meaning was conceived as “ontological significance of life from the point of view of the experiencing individual” (p. 201). The general hypothesis derived from Frankl’s theory is that life meaning would be a positive predictor of psychological well-being.

There are other conceptualizations of meaning of life in the scientific literature. Heintzelman (2018) proposed that meaning of life has three aspects: “purpose, significance, and coherence,” with purpose meaning pursuing significant goals which makes one feel “life is worth living”; significance refers to making significant contributions transcending the self; coherence means that the “stimuli, events, and one’s life make sense” (p. 7). George and Park (2016) proposed a similar “tripartite view” of meaning of life that has three components. The first component is “comprehension” which has similar meaning with “coherence”; the second component is “purpose” referring to having a valuable goal and clear direction in one’s life; the third component is “mattering” which refers to a feeling that one’s existence in the world is “of significance, importance, and value” (George and Park, 2016, p. 206). Steger et al. (2009) proposed that life meaning contains two dimensions, including “presence of life meaning” (i.e., “having” meaning) and “search for life meaning” (i.e., “seeking” of meaning). Regarding “presence of life meaning,” it refers to whether one sees one’s life as important, valuable, with clear direction, purposeful, and meaningful. On the other hand, “seeking” life meaning is a process through which one identifies or works out how one can have a meaningful life. Generally speaking, while a positive relationship was identified between “presence of life meaning” and psychological well-being, the relationship between “search for life meaning” and mental health is more complex. For example, the study of Steger et al. (2008) suggested that the relationships among these dimensions of life meaning may differ across cultures. Furthermore, Ryff (2013, p. 235) argued that in contrast to “hedonic” wellbeing, meaning of life belongs to the “eudaimonic” approach of wellbeing which involves a “reconstruction process” to “make things interpretable.” While there are differences in these theories of meaning of life, they all highlighted and indicated that meaning of life would contribute to an individual’s positive development and mental health.

With specific reference to young people, the importance of meaning in life is strongly emphasized in youth development models. For example, the Search Institute proposed 20 internal and 20 external developmental assets for the optimal development of adolescents^1^. Under “positive identity,” there is an asset of “sense of purpose” which refers to whether young people perceive their lives as purposeful. In another framework on positive youth development (PYD) attributes, based on reviewing a large amount of PYD programs, Catalano et al. (2004) found 15 PYD attributes underlying successful PYD programs. Among the 15 PYD constructs, spirituality is listed as an attribute shaping effective PYD programs. Spirituality was defined as “relating to, consisting of, or having the nature of spirit; concerned with or affecting the soul; of, from, or relating to God; of or belonging to a church or religion” (Catalano et al., 2004, p.105). Furthermore, with reference to the models focusing on the importance of spirituality strengths (e.g., Lerner, 2004; Starnino et al., 2012), life meaning is defined as “inner strength” which could promote positive well-being and reduce psychosocial adjustment problems in adolescents (Lin and Shek, 2019). As remarked by Krok (2018), “purpose embedded in the concept of meaning in life appears central to the formation of adolescent well-being as young people come to establish overarching aims” (p. 96).

### Meaning in life, anxiety and depression

Regarding the negative relationship between anxiety and meaning in life, there are at least two possible explanations. First, a high level of anxiety may make a person difficult to appreciate meaning in life because anxiety would make a person exhausted and confused. Second, the lack of life meaning is anxiety-provoking, as proposed in logotherapy that existential vacuum leads to “noogenic neurosis.” Empirically, there are studies supporting the negative relationship between anxiety and life meaning. In a study based on 1,538 German participants, Schnell and Krampe (2020) revealed that life meaning was negatively associated with mental distress indexed by anxiety and depression. They also found that life meaning moderated the impact of COVID-19 stress and mental distress. In another study involving 202 Dutch old people, Korte et al. (2012) reported a negative association among life meaning, anxiety and depression, and life meaning served as a mediator between reminiscence and depression. However, although the relationship between life meaning and anxiety is negative when using a unidimensional measure of life meaning, the relationship is complex if we look at different dimensions of meaning in life. For instance, Steger et al. (2009) found that while there was a negative linkage between “presence of life meaning” and anxiety, the relationship between “search for life meaning” and anxiety was positive.

Regarding the relationship between life meaning and depression, studies showed a negative linkage between purpose in life and depression, or life meaning is a predictor of depression (i.e., the direct effect of life meaning on depression). Based on 401 young men, Kleftaras and Psarra (2012) found that higher life meaning was related to lower depression and different dimensions of life meaning were related to different levels of depression. They concluded that “those with higher meaning of life present a better psychological health” (p. 337). Utilizing a large sample based on adult participants, Schaefer et al. (2013) investigated the association between purpose in life and emotional recovery. They identified that higher purpose in life predicted better emotional recovery after demographic variables and initial emotional reactivity were controlled. They reasoned that purpose in life is a factor protecting a person from negative life events through more resilient emotional regulation and emotional provocation.

Clinical studies also showed a negative linkage between life meaning and depression. Based on adult patients, Doolittle and Farrell (2004) found that spiritual involvement, spiritual beliefs and physical health were predictors of depression. Dursun et al. (2022) compared people with general anxiety disorders (*N* = 38) and control participants (*N* = 31) and found that participants with general anxiety disorders showed higher death anxiety and lower presence of life meaning and hardiness; life meaning was also a predictor of death anxiety. Furthermore, the negative linkage between life meaning and depression is also exhibited in adolescents. In a study of 215 university students, Hedayati and Khazaei (2014) showed negative relationships between depression and life meaning (including “presence” and “search for” meaning). Parra (2020) also reported a moderate negative linkage of the meaning of life with depression under the pandemic.

Besides the negative effect of life meaning on depression, other studies showed the protective and moderating effect of meaning in life on depression. In a longitudinal study over 2.5 years involving 909 African Americans, Park et al. (2020) showed that life meaning predicted a reduction in depression and an increase in positive emotions over time, and then they concluded that “meaning in life appears to robustly protect against future depressive symptoms and promote positive affect over time” (p. 3037) and such protective effect was not affected by demographic factors and the related stress experience. Based on two large national samples (*N* = 3,664), Hartanto et al. (2020) examined the relationships among purpose in life, child abuse and depression. Results revealed that purpose in life moderated the impact of emotional abuse in childhood on depression in adulthood. They highlighted the important role of life purpose in “building resilience, coping against adverse life events, and psychological well-being” (p. 473).

Researchers have also identified the moderating effect of life meaning in young people. Based on 204 university students in Slovakia, Halama and Bakosova (2009) showed that the overall sense of life meaning was a moderator of perceived stress with avoidance coping but not with emotional coping. Basu et al. (2022) also reported that meaning-making moderated the impact of traumatic life events experienced and suicidal ideation in 568 undergraduate students. Finally, for a sample of 177 adolescents, Dulaney et al. (2018) found a negative association between life meaning and depression symptoms, and life meaning moderated the impact of stress exposure on depression.

The moderating function of life meaning on depression is also found in the clinical field. With 151 helping professionals, Chan et al. (2021) examined the relationships among self-competence in death work, life meaning, and depressive symptoms. They showed significant correlations among these three measures, and life meaning moderated the association between depressive symptoms and self-competence in death work. In another study involving 49 psychiatric patients, Heisel and Flett (2004) showed that purpose in life had a positive association with satisfaction with life, and a negative association with neuroticism, hopelessness and depression. Besides, purpose in life and life satisfaction explained additional variance in suicidal ideation, and purpose in life moderated the impact of depression on suicidal ideation.

Meaning in life also plays an important role under adverse environmental conditions. Based on data collected from 12,243 subjects from 30 countries, Eisenbeck et al. (2021) showed that meaning-centered coping was negatively related to stress and psychological morbidity indexed by anxiety and depression symptoms during the pandemic; meaning-making coping also moderated the impact of risk factors on psychological morbidity, including depression. They concluded that life meaning plays a “critical role of meaning-centered coping in attenuating the detrimental effects of the COVID-19 pandemic on psychological distress, especially on depressive symptoms” (p. 9).

Nevertheless, some studies did not support the protective effects of life meaning. Hedberg et al. (2010) collected data from 189 old persons. While the depressive elderly scored lower on purpose in life, purpose did not predict depression 5 years later. Based on 90 patients, Kim et al. (2019) revealed that while life purpose mediated the impact of depression on quality of life, no moderating effect was found. Also, there are studies founding that the association between life meaning and mental well-being depends on the aspects of meaning in life. With 753 participants, Yek et al. (2017) found that presence of meaning was negatively linked with health anxiety while meaning search functioned oppositely, and further, higher meaning search and higher meaning presence reduced health anxiety than higher search and lower presence of meaning.

There are several gaps in the scientific literature on the relationship between meaning in life and depression in adolescents. First, although life meaning is an important developmental domain in adolescents, there is insufficient research (Zhu et al., 2022). Second, in their review of constructs of PYD used in effective programs on PYD, Catalano et al. (2004) found that only very few effective programs incorporated spirituality in the interventions. Third, most of the studies in this field are Western studies. There are two arguments for why we need more Chinese studies. The first reason is that because of the huge number of Chinese adolescents, we need more studies to ascertain the generalizability of Western theories. The second reason is that as Confucian thoughts place a strong emphasis on life meaning, it is theoretically interesting to understand the linkage between life meaning and well-being in Chinese adolescents. Fourth, in the related studies in the field, the sample size was generally small, hence limiting the generalizability of the related results. Fifth, longitudinal studies are few in the field, hence limiting our ability to understand the causal linkage between life meaning and psychological well-being. Sixth, there are few studies examining life meaning and psychological well-being within the context of COVID-19 (Zhu et al., 2022). This area is important because COVID-19 is anxiety-provoking, particularly because of changing teaching and learning modes (Shek et al., 2022a,b). As such, positive psychological attributes such as life meaning would have important functions in adjustment during the pandemic. Finally, we need more studies to clarify the moderating effects of life meaning in the context of the anxiety-depression relationship in Chinese young people (Shek, 2021).

### The present study

As there are more theories and research findings supporting the hypothesis that anxiety is an antecedent of depression, we adopted this perspective in this study. Specifically, we asked several research questions:

Research Question 1: What is the concurrent and longitudinal relationships between anxiety and depression? We predicted that anxiety would positively predict depression (Hypothesis 1).

Research Question 2: What is the relationship between anxiety and change in depression over time? Based on the literature, we expected that Wave 1 anxiety would positively predict change in depression over time (Hypothesis 2).

Research Question 3: Is life meaning concurrently and longitudinally related to depression? We expected that a negative relationship would exist between life meaning and depression (Hypothesis 3).

Research Question 4: Is life meaning related to change in depression over time? Based on the previous studies, we expected that Wave 1 life meaning would be a negative predictor of change in depression over time (Hypothesis 4).

Research Question 5: Does meaning in life moderate the influence of anxiety on depression? We expected that there would be a moderating function of life meaning on the association between anxiety and depression (Hypothesis 5).

## Materials and methods

### Participants and procedure

The participants of the present study were 4,981 students coming from five schools (two primary schools, one secondary school, and two schools enrolling both primary and secondary students) in Chengdu, mainland China. The schools agreed to join the study and formal consent was collected from the schools, students, and parents. The study adopted a longitudinal design in which the participants responded to a questionnaire at two time points. For data collection at the first time point (i.e., Wave 1: from December 2019 to January 2020 before school lockdown because of the outbreak of COVID-19), the students from the five schools responded to a questionnaire in the paper-and-pencil format in school classrooms. The study purpose and confidentiality of data and personal information were made clear to the students before they responded to the questionnaire. In total, 5,690 students responded to the questionnaire at the first wave. For the second wave (from June to July 2020 after the resumption of school), the students were invited to do the questionnaire again (*N* = 4,981). In total, 4,981 participants were matched for both waves. The mean age of the participants at Wave 1 was 13.15 ± 1.32 years old, with 48.5% were female.

### Instrument

#### Anxiety

Anxiety was examined through the “Screen for Child Anxiety Related Emotional Disorders (SCARED).” The SCARED assesses children’s anxiety disorders (Birmaher et al., 1999; Muris, 2002). It includes 41 items under five dimensions: “Panic/Somatic,” “Generalized Anxiety,” “Separation Anxiety,” “Social Phobia,” and “School Phobia.” Being validated in adolescent populations across cultures, the scale demonstrated good reliability and stable factor structure (Hale et al., 2005; Su et al., 2008; Crocetti et al., 2009). Through a three-point scale, the participants evaluated each item to indicate the level of their experiencing the specified anxiety symptom (“0” = “Never,” “1” = “Sometimes,” and “2” = “Often”). A higher score (i.e., the sum of all item scores) refers to a higher level of anxiety.

#### Depression

We used “Center for Epidemiologic Studies Depression Scale (CES-D)” to measure depression (Radloff, 1977). The CES-D includes 20 items representing 20 depressive symptoms. Each item describes a feeling or behavior related to different depressive symptoms. The participants needed to select the frequency of their having the feeling or engaging in the behavior in the past week through a scale with four points (“0” = “rarely or none of the time (<1 day),” “1” = “some or little of the time (1–2 days),” “2” = “moderately or much of the time (3–4 days),” and “3” = “most or almost all the time (5–7 days)”). The CES-D was widely used and validated across different age groups (Roberts et al., 1990; Radloff, 1991). Previous studies support the factorial validity of the Chinese CES-D (Dou et al., 2021; Zhu et al., 2021). The scale score was the sum of all items scores, with scores of four items measuring “positive affect” that were reversely coded. Higher depression was indicated by higher composite score.

#### Life Meaning

Life meaning was measured through “Spirituality” (SP) dimension of the “Chinese Positive Youth Development Scale (CPYDS).” Referring to positive youth development (PYD) indicators identified by Catalano et al. (2004), the CPYDS was developed by assessing 15 PYD attributes in Chinese youth (Shek et al., 2007). The measure showed desirable internal consistency and validity in different validation studies (Shek and Ma, 2010; Shek and Chai, 2020). The SP subscale contains seven items measuring adolescents’ meaning of life, with each item being evaluated through a seven-point scale. The composite score is generated by averaging all item scores; the higher the score is, the higher the life meaning is.

### Data analyses

Descriptive analyses were performed which include mean scores and standard deviation (SD). Besides, mean inter-item correlation and Cronbach’s *α* for all major variables were also computed. We also computed correlations between different variables. To answer Research Question 1 and Research Question 3, several hierarchical multiple regression analyses were performed to test the predicting effects of anxiety (i.e., SCARED) and life meaning (i.e., SP) on depression (i.e., CES-D) both at each wave and from the first wave to the second wave, with effects of demographic variables (i.e., age and gender) being controlled. To answer Research Question 2 and Question 4, hierarchical multiple regressions were also computed to test the predicting effect of SCARED and SP at Wave 1 on CES-D at Wave 2 with the effects of CES-D at Wave 1 being controlled. Finally, to examine whether SP moderated the predicting effect of SCARED on CES-D (i.e., Research Question 5), hierarchical multiple regression was conducted. In Step 1, we controlled age and gender effects. In Step 2, SCARED at the first wave and SP at the second wave were put in the analyses. In Step 3, we included the interaction between SCARED at the first wave and SP at the second wave. Adopting 2,000 re-samplings, we conducted bootstrapping to examine bias-corrected (BC) 95% confidence intervals (CIs) for the regression coefficients for all the hierarchical multiple regression analyses.

## Results

The descriptive statistics of major variables are shown in Table 1. The Cronbach’s alpha values for SCARED, SP, and CES-D in both waves ranged from 0.89 to 0.96, indicating excellent internal consistency. Table 2 shows correlations between different variables involved in the study in the expected directions. As both age at the first wave and gender had positive correlation with CES-D at both waves (*r* = 0.04–0.12, *ps* < 0.01). their effects were controlled in hierarchical multiple regression on the effects of SCARED and SP on CES-D.

**Table 1:** Mean, standard deviation (SD), Cronbach’s alpha, and mean inter-item correlation of different variables.

		Male *n* (%)	Female *n* (%)		
1.	Gender	2,566 (51.5%)	2,415 (48.5%)		
		**Mean**	**SD**	**Cronbach’s a**	**Mean inter-item correlation**
2.	Age (W1)	13.15	1.32		
3.	SCARED (W1)	16.97	14.84	0.95	0.31
4.	SP (W1)	5.69	1.26	0.89	0.55
5.	CES-D (W1)	14.10	10.45	0.89	0.32
6.	SCARED (W2)	15.86	15.30	0.96	0.35
7.	SP (W2)	5.50	1.40	0.93	0.65
8.	CES-D (W2)	14.76	11.07	0.91	0.36

W1 = Wave 1; W2 = Wave 2.

**Table 2:** Correlations between different variables.

	Variable	1	2	3	4	5	6	7
1.	Age (W1)	–						
2.	Gender	0.01	–					
3.	SCARED (W1)	0.09***	0.15***	–				
4.	SP (W1)	−0.18***	−0.06***	−0.48***	–			
5.	CES-D (W1)	0.12***	0.04**	0.61***	−0.62***	–		
6.	SCARED (W2)	0.08***	0.17***	0.55***	−0.39***	0.44***	–	
7.	SP (W2)	−0.12***	−0.10***	−0.40***	0.62***	−0.50***	−0.54***	–
8.	CES-D (W2)	0.12***	0.06***	0.43***	−0.47***	0.55***	0.61***	−0.64***

^***^p < 0.001; ^**^p < 0.01; W1 = Wave 1; W2 = Wave 2.

Results of the hierarchical multiple regression in Table 3 showed that SCARED significantly and positively predicted CES-D at each wave (for Wave 1 and 2, *B* = 0.43 and 0.45, 95% bootstrap confidence intervals (CIs) = [0.41, 0.45] and [0.43, 0.47], *β* = 0.61 and 0.62, *ps* < 0.001. Cohen’s *f^2^* = 0.570 and 0.594, respectively). Longitudinally, there was significant positive predicting effect of SCARED at the first wave on CES-D at the second wave (*B* = 0.32, 95% bootstrap confidence intervals (CIs) = [0.29, 0.34], *β* = 0.42, *p* < 0.001, Cohen’s *f^2^* = 0.213; Table 4). The findings give support to Hypothesis 1. In addition, SCARED at the first wave also significantly and positively predicted change in CES-D over time (*B* = 0.11, 95% bootstrap confidence intervals (CIs) = [0.08, 0.14], *β* = 0.15, *p* < 0.001, Cohen’s *f^2^* = 0.019). Table 4 shows the details of the results. Therefore, Hypothesis 2 was also supported.

**Table 3:** Cross sectional predicting effects of anxiety (SCARED) on depression (CES-D).

Model	Predictor	W1 CES-D							W2 CES-D						
		*B*	BC 95% CI		SE	*β*	*t*	Cohen’s *f^2^*	*B*	BC 95% CI		SE	*β*	*t*	Cohen’s *f^2^*
			Lower	Upper						Lower	Upper				
1	W1 Age	0.92	0.71	1.13	0.11	0.12	8.20***	0.014	0.98	0.74	1.22	0.12	0.12	8.32***	0.014
	Gender	0.83	0.25	1.44	0.31	0.04	2.79**	0.002	1.32	0.68	1.93	0.32	0.06	4.24***	0.004
	*R^2^* change	0.015							0.017						
	*F* change	37.620***							43.768***						
2	SCARED	0.43	0.41	0.45	0.01	0.61	52.93***	0.570	0.45	0.43	0.47	0.01	0.62	54.38***	0.594
	*R^2^* change	0.357							0.366						
	*F* change	2801.316***							2957.195***						

^***^p < 0.001; ^**^p < 0.01; W1 = Wave 1; W2 = Wave 2.

**Table 4:** Longitudinal predicting effects of anxiety (SCARED) on depression (CES-D) and its change.

Model	Predictor	W2 CES-D							Predictor	W2 CES-D						
		*B*	BC 95% CI		SE	*β*	*t*	Cohen’s *f^2^*		*B*	BC 95% CI		SE	*β*	*t*	Cohen’s *f^2^*
			Lower	Upper							Lower	Upper				
1	W1 Age	0.99	0.77	1.21	0.12	0.12	8.32***	0.014	W1 Age	0.99	0.76	1.23	0.12	0.12	8.32***	0.014
	Gender	1.33	0.71	1.93	0.32	0.06	4.24***	0.004	Gender	1.33	0.68	1.97	0.32	0.06	4.24***	0.004
	*R^2^* change	0.017							*R^2^* change	0.017						
	*F* change	43.741***							*F* change	43.741***						
2	W1 SCARED	0.32	0.29	0.34	0.01	0.42	32.57***	0.213	W1 CES-D	0.58	0.55	0.61	0.02	0.55	45.76***	0.426
	*R^2^* change	0.174							*R^2^* change	0.293						
	*F* change	1,060.799***							*F* change	2,093.559***						
3									W1 SCARED	0.11	0.08	0.14	0.01	0.15	9.75***	0.019
									*R^2^* change	0.013						
									*F* change	95.024***						

^***^p < 0.001; W1 = Wave 1; W2 = Wave 2.

In Table 5, results showed that SP significantly and negatively predicted CES-D (for Wave 1 and Wave 2, *B* = −5.12 and −5.04, 95% bootstrap confidence intervals (CIs) = [−5.37, −4.86] and [−5.26, −4.82], *β* = −0.62 and −0.64, *ps* < 0.001. Cohen’s *f^2^* = 0.593 and 0.680, respectively). SP at the first wave also negatively predicted CES-D at the second wave (*B* = −4.07, 95% bootstrap confidence intervals (CIs) = [−4.34, −3.79], *β* = −0.46, *p* < 0.001, Cohen’s *f^2^* = 0.266; Table 6). This provides support for Hypothesis 3. Besides, after controlling the effect of first-wave CES-D, first-wave SP negatively predicted second-wave CES-D (*B* = −1.77, 95% bootstrap confidence intervals (CIs) = [−2.07, −1.47], *β* = −0.20, *p* < 0.001, Cohen’s *f^2^* = 0.037). This provided support for Hypothesis 4.

**Table 5:** Cross sectional predicting effects of life meaning (SP) on depression (CES-D).

Model	Predictor	W1 CES-D							W2 CES-D						
		*B*	BC 95% CI		SE	*β*	*t*	Cohen’s *f^2^*	*B*	BC 95% CI		SE	*β*	*t*	Cohen’s *f^2^*
			Lower	Upper						Lower	Upper				
1	W1 Age	0.92	0.70	1.15	0.11	0.12	8.20***	0.014	0.98	0.75	1.21	0.12	0.12	8.32***	0.014
	Gender	0.83	0.28	1.40	0.30	0.04	2.79**	0.002	1.32	0.72	1.92	0.31	0.06	4.24***	0.004
	*R^2^* change	0.015							0.017						
	*F* change	37.620***							43.768***						
2	SP	−5.12	−5.37	−4.86	0.12	−0.62	−53.99***	0.593	−5.04	−5.26	−4.82	0.11	−0.64	−58.18***	0.680
	*R^2^* change	0.366							0.398						
	*F* change	2,914.498***							3,385.299***						

^***^p < 0.001; ^**^p < 0.01; W1 = Wave 1; W2 = Wave 2.

**Table 6:** Longitudinal predicting effects of life meaning (SP) on depression (CES-D) and its change.

Model	Predictor	W2 CES-D							Predictor	W2 CES-D						
		*B*	BC 95% CI		SE	*β*	*t*	Cohen’s *f^2^*		*B*	BC 95% CI		SE	*β*	*t*	Cohen’s *f^2^*
			Lower	Upper							Lower	Upper				
1	W1 Age	0.99	0.77	1.20	0.12	0.12	8.32***	0.014	W1 Age	0.99	0.77	1.22	0.12	0.12	8.32***	0.014
	Gender	1.33	0.70	1.94	0.32	0.06	4.24***	0.004	Gender	1.33	0.65	1.99	0.33	0.06	4.24***	0.004
	*R^2^* change	0.017							*R^2^* change	0.017						
	*F* change	43.741***							*F* change	43.741***						
2	W1 SP	−4.07	−4.34	−3.79	0.14	−0.46	−36.18***	0.266	W1 CES-D	0.58	0.55	0.61	0.02	0.55	45.76***	0.426
	*R^2^* change	0.207							*R^2^* change	0.293						
	*F* change	1,309.311***							*F* change	2,093.559***						
3									W1 SP	−1.77	−2.07	−1.47	0.16	−0.20	−13.47***	0.037
									*R^2^* change	0.025						
									*F* change	181.490***						

^***^p < 0.001; W1 = Wave 1; W2 = Wave 2.

**Figure 1:**
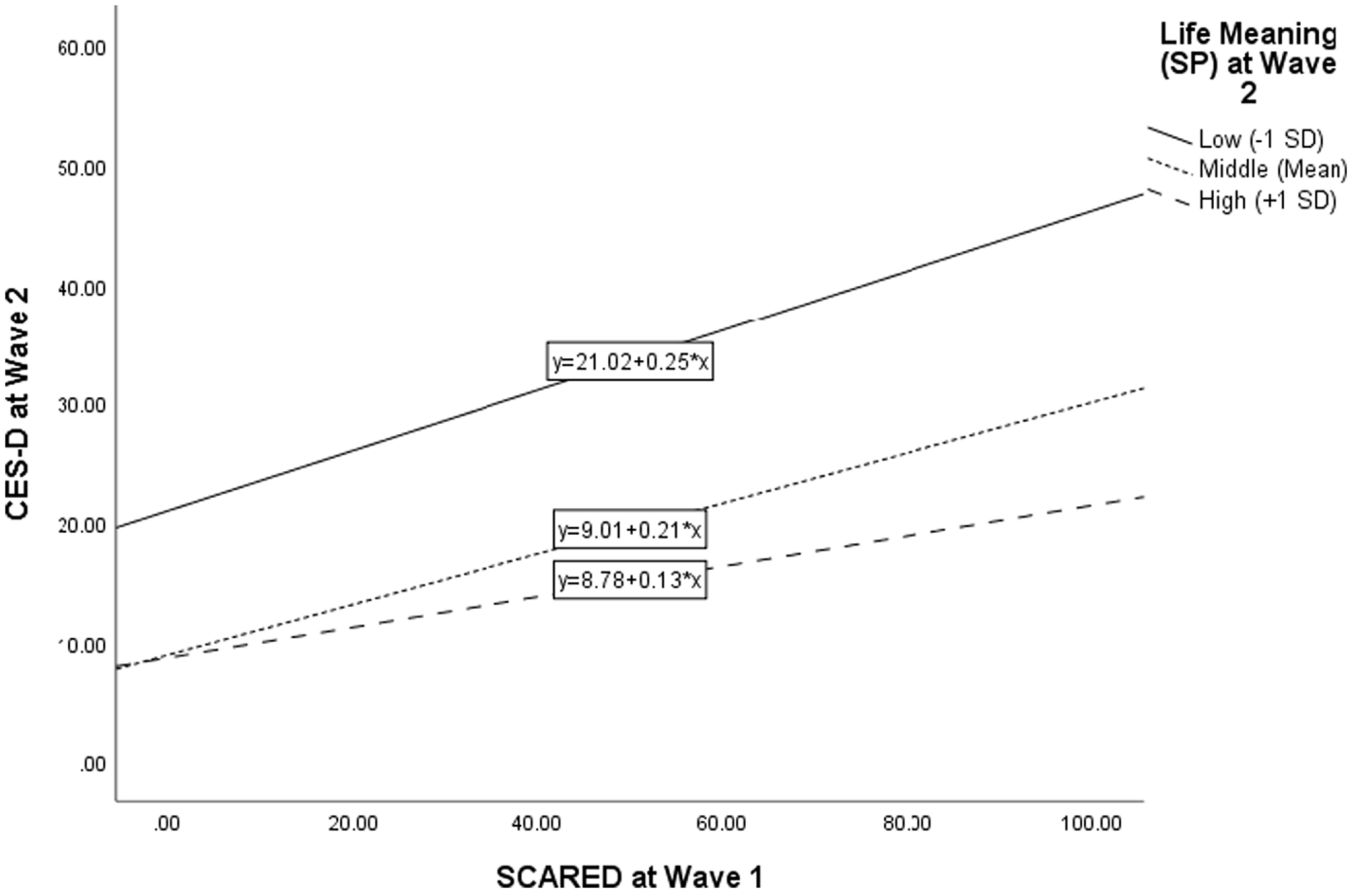
Moderating effect of SP at Wave 2 on the relationship between SCARED at Wave 1 and CES-D at Wave 2.

Another hierarchical regression was conducted to test the moderating effect of SP on the predicting effect of SCARED on CES-D (Table 7). In the first step, the effects of demographic variables were controlled. In the second step, predictors of SCARED at Wave 1 and SP at Wave 2 were put in the regression model. Results showed significant main effects of the first-wave SCARED (*B* = 0.16, 95% bootstrap confidence intervals (CIs) = [0.14, 0.18], *β* = 0.21, *p* < 0.001, Cohen’s *f^2^* = 0.067) and the second-wave SP (*B* = −4.42, BC 95% CI = [−4.66, −4.19], *β* = −0.56, *p* < 0.001, Cohen’s *f^2^* = 0.478) on the second-wave CES-D. In Step 3, the interaction between the first-wave SCARED and the second-wave SP was put in the analyses. The interaction between the first-wave SCARED and the second-wave SP had a significant predicting effect on the second-wave CES-D (*B* = −0.71, 95% bootstrap confidence intervals (CIs) = [−0.96, −0.46], *β* = −0.08, *p* < 0.001, Cohen’s *f^2^* = 0.010). This suggests the predicting effect of SCARED on CES-D was moderated by SP, hence supporting Hypothesis 5. The analyses of the simple slopes (Figure 1) showed that the predicting effect of the first-wave SCARED on the second-wave CES-D was more positive for participants with lower SP (−1 SD; *β* = 0.36, *p* < 0.001) than the participants with higher SP (+1 SD; *β* = 0.22, *p* < 0.001) at the first wave. The findings also provided support for Hypothesis 5.

**Table 7:** Hierarchical multiple regression analyses for the predicting effects of anxiety (SCARED) at Wave 1 and life meaning (SP) at Wave 2 on depression (CES-D) at Wave 2.

Model	Predictor	*B*	BC 95% CI		SE	*β*	*t*	Cohen’s *f^2^*	*F* change	*R^2^* change
			Lower	Upper						
1	W1 Age	0.99	0.74	1.23	0.12	0.12	8.324***	0.014	43.74***	0.017
	Gender	1.33	0.69	1.95	0.32	0.06	4.243***	0.004		
2	W1 SCARED	0.16	0.14	0.18	0.01	0.21	18.093***	0.067	1958.71***	0.436
	W2 SP	−4.42	−4.66	−4.19	0.12	−0.56	−48.476***	0.478		
3	W1 SCARED**×**W2 SP	−0.71	−0.96	−0.46	0.13	−0.08	−7.139***	0.010	50.959***	0.006

^***^p < 0.001; W1 = Wave 1; W2 = Wave 2.

## Discussion

The study has several advances concerning the existing research gaps. First, as research on meaning in life has predominantly been done in adult samples, we recruited adolescent samples for the present study. Second, in view of the scarcity of Chinese studies on meaning in life, we recruited Chinese students for the present study. The findings can give some insight into the applicability of Western theories and research findings in the Chinese context. Third, in contrast to the common practice of recruiting small samples, we employed a large sample in this study. Fourth, utilizing two waves of data, we can look at the concurrent as well as longitudinal relationships between anxiety (and life meaning) and depression. Fifth, we examined the relationship among anxiety, life meaning and depression within the context of COVID-19 pandemic which is pioneered in the field. Sixth, we tested the moderating function of life meaning in the predictive relation between anxiety and depression.

Hypothesis 1 was supported with results showing that anxiety concurrently and longitudinally predicted depression in Chinese adolescents in the context of the pandemic. Also, supporting Hypothesis 2, anxiety predicted the change in depression from the first to the second wave. The findings are in line with the existing empirical results suggesting that anxiety could be an antecedent of depression (e.g., Cole et al., 1998; Chaplin et al., 2009; Price et al., 2016). For instance, a study based on two-wave data showed that anxiety symptoms predicted depressive symptoms in middle school students 1 year later (Chaplin et al., 2009). Another six-wave longitudinal study based on both student-and parent-report data identified that early-time anxiety predicted later-time change in depression in primary school students (Cole et al., 1998). The results provide further empirical support to the theoretical view that anxiety is a precursor or risk factor for depression (Mathew et al., 2011). Cummings et al. (2014) also explained that adolescents with a constitutional predisposition to anxiety tended to develop “anxiety-related impairment” if their anxiety remains untreated, and the “anxiety-related impairment” would be a risk factor for depressive symptoms.

Supporting Hypothesis 3, this study showed that meaning in life both concurrently and longitudinally negatively predicted depression. The results are in line with the existing empirical studies suggesting the negative linkage of life meaning with depression based on the general population and clinical samples (Doolittle and Farrell, 2004; Kleftaras and Psarra, 2012; Schaefer et al., 2013). Also, supporting Hypothesis 4, the study showed that life meaning negatively predicted change in depression. The findings extend the existing literature showing that life meaning is a unique negative predictor of depressive symptoms in Chinese youth during the pandemic. This highlights the important role of life meaning in decreasing the risk for depressive symptoms. In addition, the results provide support to Frankl’s theory of life meaning that individuals are not motivated by “will to pleasure” but are driven by a meaning in life (Frankl, 1959; Shek, 1992).

Findings of the study also support Hypothesis 5, showing that meaning in life moderated the predictive effect of anxiety on depression. This is in line with the existing literature that persons having higher meaning in life would develop lower depression compared with persons with lower meaning in life when confronting the same level of negative or stressful life events (Hartanto et al., 2020; Park et al., 2020). However, many of the existing studies were based on adult or general population samples, there were few studies on youth or adolescents. Also, there were few studies on Chinese adolescents. Whether life meaning functioned as a protector in Chinese youth against mental health problems under stressful environments was not very clear (Zhang et al., 2018). This study provides evidence of the important protective function of life meaning in Chinese culture. Besides, the results shed light on the unique role of life meaning in maintaining mental health under the COVID-19 pandemic, which posed significant stress to individuals, particularly adolescents who are more vulnerable to stress. There are consistent reports of an increased level of mental health problems such as depression in adolescents during COVID-19 (Miranda et al., 2020; Hawes et al., 2021). The present research echoes the claim that life meaning could be an important buffer against mental health problems.

The present study has several theoretical implications. First, it offers further empirical support for the role of anxiety being a precursor or antecedent of depressive symptoms (Cohen et al., 2018). Particularly, it provides support to the theoretical proposition that anxiety could be a causal factor for depression because its impairment would lead to comorbid or more severe mental health problems (Cummings et al., 2014). Second, the study provides support to the protective function of life meaning in the mental health of adolescents under stressful situations. This further confirms and highlights the important role of life meaning in positive youth development models in literature (e.g., Catalano et al., 2004; Benson, 2007).

The study also has several practical implications. First, as anxiety could be a precursor of depression, it is highly important to assess and treat anxiety in young people to prevent the later onset of depressive symptoms. Second, as life meaning plays important role in reducing depression and moderating the predicting effect of anxiety on depression, it is also key to promote the life meaning of adolescents through different educational or intervention programs. Third, the present study also indicates the importance of implementing PYD programs in adolescents during the pandemic period to promote their mental health. Unfortunately, there are few related studies under the pandemic (Shek, 2021; Shek et al., in press).

Several limitations of the study should be noted. First, as the study was only based on data collected from two waves, the longitudinal predicting role of anxiety in depression should be further tested with data collected from more waves. Second, the participants of the study were recruited from five schools. While the schools were randomly selected, further studies should be performed based on participants from more schools to verify the results and increase the generalizability of the results. Third, we used a unidimensional measure to assess life meaning. As life meaning may contain multiple dimensions, future studies should test the moderating role of life meaning based on the multidimensional measure. Despite of these limitations, the study provides support to the risk role of anxiety and the protective role of life meaning in depression in Chinese adolescents, which shed light on the theoretical understanding of the relationship between these mental health factors and on the prevention in mental health among adolescents in China during the pandemic.

## Data availability statement

The raw data supporting the conclusions of this article will be made available by the authors, without undue reservation.

## Ethics statement

The studies involving human participants were reviewed and approved by the Ethics Committee of Sichuan University. Written informed consent to participate in this study was provided by the participants’ legal guardian/next of kin.

## Author contributions

DS designed the research project and contributed to all the steps of the work. WC conducted data analyses and contributed to the first draft, and revised the manuscript based on the comments and editing provided by DS. LT contributed to the drafts at different stages and proof-read the paper. All authors contributed to the article and approved the submitted version.

## Funding

This work is financially supported by Wofoo Foundation and the Research Matching Fund of the Research Grants Council (R.54.CC.83Y7).

## Conflict of interest

The authors declare that the research was conducted in the absence of any commercial or financial relationships that could be construed as a potential conflict of interest.

## Publisher’s note

All claims expressed in this article are solely those of the authors and do not necessarily represent those of their affiliated organizations, or those of the publisher, the editors and the reviewers. Any product that may be evaluated in this article, or claim that may be made by its manufacturer, is not guaranteed or endorsed by the publisher.

## Footnotes

^1^ https://searchinstitute.org/
